# Facilitators and barriers to the acceptance of human papillomavirus (HPV) vaccination among adolescent girls: a comparison between mothers and their adolescent daughters in Hong Kong

**DOI:** 10.1186/s13104-017-2734-2

**Published:** 2017-08-10

**Authors:** Alice Yuen Loke, Ava Chiu Oi Chan, Yuen Ting Wong

**Affiliations:** 1 School of Nursing, The Hong Kong Polytechnic University, Hung Hom, Kowloon, Hong Kong; 2 The Hong Kong Sanatorium and Hospital, Happy Valley, Hong Kong

**Keywords:** Human papillomavirus (HPV) vaccination, Receptiveness to vaccination, Mother–daughter dyads

## Abstract

**Background:**

The aim of this study is to examine knowledge and attitude as facilitators and barriers to the acceptance of HPV vaccination for adolescent girls by mothers and adolescent girls.

**Methods:**

A cross-sectional survey conducted in Hong Kong in January 2010. Adolescent girls aged 12–18, together with their mothers, were recruited to complete two separate questionnaires with similar questions.

**Results:**

A total of 170 mother–adolescent girl dyads were recruited. When the daughters and mothers were compared, the mothers were found to be more aware of “the risk of becoming infected with HPV through early sexual intercourse,” while more daughters than mothers knew that “the HPV virus cannot be cured with antibiotics.” Significantly more daughters perceived that they had a “chance of being infected with HPV and getting cervical cancer without the vaccine,” while more mothers were concerned that “vaccinating for HPV will cause a girl to be stigmatized as promiscuous” and thought that their “adolescent daughters are too young to receive the HPV vaccine.” The major predictive factor for the acceptance of the HPV vaccine among mothers was “The HPV vaccine is safe” (OR = 10.126, 95% CI 2.47–41.54). Among daughters who accepted the vaccine, the predictive factor was “The HPV vaccine can prevent most HPV infections” (OR = 6.274, 95% CI 1.93–20.42).

**Conclusions:**

The findings provide healthcare professionals with a better understanding of the differences between mothers and adolescent girls in knowledge, attitude, and potential factors associated with acceptance of the HPV vaccine. Health professionals should promote the early prevention of HPV infection and eliminate the stigma surrounding HPV vaccination to increase its acceptance. The government should provide financial support for adolescent girls to receive the vaccination in school.

## Background

Cervical cancer is the second most common form of cancer among women worldwide. According to the World Health Organization (WHO), every year almost 270,000 women die of cervical cancer and over 530,000 new cases of cervical cancer are reported [1]. In Hong Kong, cervical cancer was the eighth most common form of cancer and the ninth most common cause of mortality from cancer among females in 2012 [2]. The lifetime risk among Hong Kong Chinese women of contracting cervical cancer is 1 in 128 [2]. Although the age-standardized incidence of cervical cancer in Hong Kong is declining at an average rate of 4% per year, the burden of the disease remains moderately high in comparison to that in Western countries of comparable economic status, such as Canada and Australia [3].

### Prophylactic human papillomavirus vaccines and recommendations

Prophylactic human papillomavirus (HPV) vaccines, which were developed in the mid-2000s, are said to prevent mostly HPV types 16 and 18, which contribute to the development of 66% of cervical cancer cases [4]. According to the Advisory Committee on Immunization Practices (ACIP) in the United States, HPV vaccination is recommended for adolescent girls aged 11–12 [5]. The recommendation for mandatory immunization of girls of school age has been based on a decision model showing that this would result in decreased morbidity and mortality from cervical cancer, and would be cost-effective when compared to other generally acceptable health interventions such as the Pap test and treatments for squamous intraepithelial lesions (SIL) and cervical cancer [6].

Some Western countries, such as the United Kingdom, have launched a national program of routine HPV vaccinations, whereby the first dose can be given to children at any time during the eighth grade [7]. Although the Hong Kong Medical Association (HKMA) also recommended back in 2008 that prophylactic HPV vaccines be included in vaccination schemes for children over the age of nine [8], Hong Kong has not established a policy of universal HPV vaccination for young girls. In Hong Kong, HPV vaccination is self-financed and voluntary [3]. The rate of acceptance of HPV vaccination among women with adolescent daughters is about 32% in Hong Kong, which is lower than in Western countries [9]. In the United States (California) and Finland, for example, this rate is 75 and 86%, respectively [10, 11].

### Acceptance of HPV vaccination among women and adolescents

Numerous studies have been conducted among women and adolescent girls since HPV vaccines became available. Acceptance of HPV vaccination has been reported to be associated with knowledge of the virus and the vaccine, its link with promiscuity and sexually transmitted infections, and the safety and effectiveness of the vaccine.

Among women, knowledge and awareness of the virus and the vaccine has been associated with their intention to be vaccinated [12]. A systematic review of studies conducted in the US, Europe, Canada, Australia, and Africa revealed that the majority of women and men had inadequate knowledge or misconceptions about the connection between the virus and cervical cancer, making it difficult for people to accept the vaccine [13]. In Hong Kong, almost all adolescents aged 13–20 in a small sample study (n = 64) were unaware of the virus and its link to HPV infection and cervical cancer [14].

Adolescents and young adults who were aware of the risks and severity of HPV infection and cervical cancer tended to be more accepting of the HPV vaccine [13, 15]. In a qualitative study that explored parents’ acceptance of HPV vaccines for their children, it was revealed that parents who had previously contracted HPV infections were more accepting of the idea of getting their children vaccinated [16]. In Hong Kong, mothers were found to be more receptive of HPV vaccination for their daughters if they considered their adolescent daughters to be susceptible to sexually transmitted infections (STI) [9]. Another study revealed that adolescents between the ages of 13 and 20 who were interviewed perceived that they were at a low risk of becoming infected with HPV and did not foresee themselves as engaging in sex in the near future [14].

### Barriers to HPV vaccination

The link between HPV and STIs is a barrier to the acceptance of HPV vaccination [17, 18]. Studies have reported that those with multiple sexual partners are more likely to form the intention to be vaccinated against HPV [12]. An early sexual debut at between the ages of 16 and 20 also increases the intention of Chinese adolescent girls to be vaccinated [19]. However, the link between HPV and sexual activity has also caused HPV vaccination to be stigmatized. Personal and parental disapproval of HPV vaccination arises from the stigma associated with STIs and the implication that allowing their child to be vaccinated is the same as giving permission to their child to engage in risky sexual behavior [20]. A systematic review of relevant studies also showed that 6–12% of parents were concerned that vaccinating adolescents against HPV would encourage early sexual behavior [13].

Parents also consider the safety and effectiveness of the HPV vaccine when making a decision on whether or not to accept HPV vaccination for their adolescent daughters [18, 21, 22]. Women worry about the potential side-effects of the vaccines and the possible adverse effects on fertility and overall health [23, 24]. Uncertainly over the duration of the efficacy of the vaccine is another consideration [14].

The high monetary cost of the HPV vaccine also poses an obstacle to achieving wider vaccination coverage. In Hong Kong, a three-shot series of the HPV vaccine costs at least HK$3000 (around US$400). Adolescents who expressed a willingness to be vaccinated reportedly changed their minds after they learned much it would cost [14].

The successful implementation of HPV vaccination depends on what adolescent girls and their parents know about the vaccination and their attitude towards it [25]. Numerous studies have been conducted on the acceptance of the vaccine among women, adolescents, or mothers for their daughters, but none has explored differences in the knowledge and attitude of mothers and adolescent daughters towards HPV vaccination and how it affects their acceptance of the vaccine.

## Application of the knowledge, attitude, and practices (KAP) model

The knowledge, attitude, and practices (KAP) model, which is based on Bandura’s Social Learning Theory [26], specifies the relationship between knowledge and attitudes towards the HPV vaccine, and factors that help to identify predictors of the acceptance of HPV (practice). This model was adopted as the conceptual framework for this study.

## Methods

### Aim and objectives of the study

The aim of the study was to examine knowledge and attitude as facilitators and barriers to the acceptance by both mothers and adolescent girls of HPV vaccination for adolescent girls. The specific objectives of this study were to explore the following issues and make comparisons between the mothers and their adolescent daughters: (1) their knowledge and attitude towards HPV and the HPV vaccine, (2) the reasons for their acceptance or non-acceptance of HPV vaccination, and (3) the factors predicting their acceptance of HPV vaccination.

### Design and setting of the study

This was a cross-sectional study, using a self-administered questionnaire. The study was conducted in one secondary school in a district (Kwai Chung) in Hong Kong in January 2010.

### Target population

Adolescent girls (aged 12–18) studying in secondary schools, together with their mothers, were the target population.

### Instrument

Two sets of questionnaires were developed—one specifically for mothers and the other for adolescent girls. The two sets of questionnaires contain identical items, except for the section on the demographic information of the mothers and daughters.

Each questionnaire consists of four parts. Part I solicits information on the demographics of the mothers or the adolescent girls. Part II contains 11 true or false questions to assess the knowledge of the mothers or daughters on HPV and the HPV vaccine including: symptoms of HPV infection (4 items), the transmission of HPV (3 items), and the HPV vaccine and cures for HPV (4 items). Part III consists of 15 statements on attitudes towards HPV infection and the HPV vaccine including: the perceived risk and severity of HPV infection and cervical cancer (4 items), stigmatization (3 items), the perception that adolescence is a young age to receive a vaccine associated with sexually transmitted diseases (1 item), the perceived safety and effectiveness of the HPV vaccine (4 items), and the accessibility of the HPV vaccine (3 items). Part IV contains 19 possible reasons related to the acceptance of HPV vaccination (1 item), and reasons for accepting (6 items) and not accepting the vaccination for HPV (12 items). Four-point Likert scales (strongly disagree to strongly agree, or very unlikely to very likely) are employed in Parts III and IV.

The items in the questionnaire were developed based on questions from the Carolina HPV Immunization Attitude Scale [27], which had been used in the following studies: the Caregiver Survey of the Carolina HPV Immunization Measurement and Evaluation (CHIME) Project [28], and the Person County pilot study on HPV vaccination [29]. All of the factors in the questionnaire for this current study were shown to have acceptable scale alphas and a 1-year test–retest reliability.

### Validity

An expert panel consisting of two gynecologists and one pediatrician, who were knowledgeable and experienced in a related field, were invited to validate the two sets of questionnaires for use in Hong Kong before implementation. All three agreed on the relevance of the items to the aim and objectives of the study. The content validity index was at 0.84 and 0.87 for the questionnaires of the mothers and daughters respectively, indicating an acceptable level of validity for the items. Minor modifications were also made on the basis of feedback from the experts.

The drafted questionnaires were piloted on seven adolescent girls (aged from 12 to 18) and their mothers by convenience sampling. The wording of the questionnaire was modified based on the comments of the girls and mothers, so that it would be easier to read and understand.

### Ethical considerations

Before embarking on the study, ethical approval was obtained from the university’s ethics committee. Permission to collect data was sought from the principal of the secondary school in Kwai Chung. Information sheets explaining the purpose of the study were distributed to the students and their mothers. The questionnaires were completed anonymously and on a voluntary basis. The adolescents who completed the questionnaires were doing so in the presence of their mothers in the School hall. The return of the completed questionnaires by mother–daughter dyads was considered as implied consent. Only the adolescents whose mothers also completed the questionnaires were included in this report.

### Data collection and analysis

The questionnaires were distributed by the researcher in a hall of the school, and collected before a scheduled talk on ‘HPV and vaccines’ was to be given to female students and their mothers. The mother–daughter dyads were invited to take part in the health talk on a voluntary basis. In order to avoid influencing the results of the study, the adolescent girls and their mothers were asked to complete the questionnaires separately without discussing them. Those adolescent girls whose mothers were not present were given the questionnaires to bring home for their mothers to complete. The completed questionnaires were to be returned within a week in a box provided in the school.

All of the data were entered into a computer using the Statistical Package for the Social Sciences (SPSS) (Version 20.0; SPSS, Chicago, Illinois, USA). Descriptive statistics were used to describe the demographics of the participants, and their knowledge, attitude, and reasons for accepting or not accepting HPV and the HPV vaccine. A Chi square test was used to compare differences in all of the included variables (with the exception of demographics) between the mothers and their adolescent daughters. Logistic regression was performed to identify factors predicting acceptance of the HPV vaccine by the mothers and daughters. The level of significance was set at *p* ≤ 0.05 for all inferential statistical analyses.

## Results

A total of 220 dyads of mothers and adolescent girls were approached. Of these, 170 mothers (response rate: 77.3%) and 211 adolescent girls (response rate: 95.9%) completed and returned the questionnaires. Completed questionnaires from adolescent girls whose mothers did not also complete their own questionnaires were discarded. In the end, only 170 mothers and 170 adolescent daughters as dyads were included in the analysis.

### Demographic characteristics of the mothers and their daughters

The demographic characteristics of the mothers and their daughters are shown in Table 1. Over half of the mothers (62.4%) were 41–50 years old. The majority of them were married (78.2%) and working in non-health related occupations (88.2%). The majority of the mothers (76.4%) had attained a secondary level of education or above, and one-third (36.5%) had a monthly family income of above HK$15,000. About half of the mothers (55.3%) had ever had a Pap smear test; among these, 24.5% had received abnormal Pap test results. Fourteen mothers (8.2%) had been diagnosed with cervical cancer and nine (5.3%) had undergone an operation for cervical cancer. Around 7.1% of the mothers indicated that they had a family history of cervical cancer.

**Table 1:** Demographics of mother and daughter dyads (N = 170 dyads)

	N	(%)
Demographics and gynecological history of the mothers (n = 170)		
Age (years)^a^		
≤30	11	6.5
31–40	28	16.5
41–50	106	62.4
≥51	24	14.1
Marital status^a^		
Married	133	78.2
Single/divorced/separated	26	15.3
Widowed	10	5.9
Occupation^a^		
Health professionals	13	7.6
Non-health related	150	88.2
Educational attainment^a^		
Primary level	39	22.9
Secondary level	108	63.5
Tertiary education and above	22	12.9
Family income (HK$)^a^		
On government assistance	10	5.9
$10,000 and below	50	29.4
$10,001–15,000	45	26.5
$15,001–30,000	36	21.2
≥$30,001	26	15.3
Most recent Pap test^a^		
Never	75	44.1
Within the last 3 years	68	40.0
More than 3 years ago	26	15.3
Past abnormal Pap test^a^ (among those who have had a Pap test, n = 94)		
Never	71	75.5
Once	11	11.7
Twice	9	9.6
Three or more	3	3.2
Ever had diagnosed with cervical cancer^a^	14	8.2
Had an operation for cervical cancer (colposcopy, total hysterectomy or loop electrosurgical excision procedure)^a^	9	5.3
Family history of cervical cancer^a^	12	7.1
Demographics and sexual experience of the adolescent daughters (n = 170)		
Age of the daughters interviewed in this study (m = 16.22 ± 1.91 years)		
12–15	44	25.9
16–18	126	74.1
Received all childhood vaccines	140	82.4
Had serious adverse effects from a childhood vaccine	7	4.1
Received the HPV vaccine	3	1.8
Self-report of sexual experience		
Never had	143	84.1
Yes, with one boyfriend	25	14.7
Yes, with multiple partners	2	1.2
Believe sexual partner has other sexual partners (among those with sexual partners) (n = 27)		
No	22	81.5
Yes	5	18.5

^a^Missing data are less than 5% (range 0.6–2.4%)

The mean age of the daughters was 16.2 years old, with 25.9% of them aged 12–15 and 74.1% aged 16–18. Most of the adolescent daughters (82.4%) had received all childhood vaccinations, and a few (4.1%) had experienced adverse effects from childhood vaccinations. Only three (1.8%) had been vaccinated against HPV. Most of the adolescent daughters reported (84.1%) that they had no sexual experience. Among those who had a sexual partner, most (81.5%) believed that their sexual partner did not have other sexual partners.

### Knowledge of HPV and the HPV vaccine among mothers and their adolescent daughters

Table 2 shows what knowledge the mothers and adolescent daughters possessed on HPV and the HPV vaccine. Chi square tests were used to compare the percentage of correct answers given by the mothers and their adolescent daughters to the statements on knowledge.

**Table 2:** Knowledge of mothers and adolescent daughters on HPV and the HPV vaccine (N = 170 dyads)

Knowledge (correct answer) on HPV and the HPV vaccine	Mothers (n = 170)		Daughters (n = 170)		χ^2^	*p* value
	n	(%)	n	(%)		
Symptoms of HPV infection						
Sores on the vagina that do not heal are one of the symptoms of HPV infection	99	58.2	72	42.4	7.502	0.006**
Warts that sometimes itch or bleed are one of the symptoms of HPV infection	85	50.0	83	48.8	0.012	0.914
Most cell changes can revert to normal and HPV infection can be cleared by bodily immunity	51	30.0	39	22.9	2.304	0.129
Persistent HPV infections can cause pre-cancer (dysplasia) and cervical cancer	115	67.6	126	74.1	1.586	0.208
Transmission of HPV						
HPV can be transmitted through sexual intercourse	116	68.2	129	75.9	2.008	0.157
Having sex with multiple sexual partners increases the risk of HPV infection	128	75.3	132	77.6	0.401	0.527
Having sex before the age of 16 increases the risk of HPV infection	105	61.8	84	49.4	5.739	0.017*
HPV vaccine and cures						
Currently, there is a way to eliminate the HPV virus (false)	67	39.4	90	52.9	7.184	0.007**
HPV can be cured with antibiotics (false)	92	54.1	119	70.0	10.756	0.001**
There is a vaccine that can protect women against cervical cancer	134	78.8	144	84.7	1.415	0.234
It is not necessary to have regular Pap smear tests after having received the HPV vaccine (false)	124	72.9	139	81.8	3.575	0.059

Individual items may or may not add up to the total due to some missing data

* *p* ≤ 0.05; ** *p* ≤ 0.01

Regarding knowledge of HPV infection, more mothers than adolescent daughters correctly indicated that “sores on the vagina that do not heal are one of the symptoms of HPV infection,” and the difference was statistically significant (58.2% vs. 42.4%, *p* = 0.006). Mothers were also more likely than their daughters to know that “warts that sometimes itch or bleed are one of the symptoms of HPV infection” (50.0% vs. 48.8%), and that “most changed cells can revert to normal and HPV infection can be cleared by bodily immunity” (30.0% vs. 22.9%), although the differences were not significant. Adolescent daughters were more likely than their mothers to know that “persistent HPV infections can cause pre-cancer (dysplasia) and cervical cancer” (67.6% vs. 74.1%), although again the difference was not statistically significant.

Both the mothers and daughters seemed to have better knowledge of the transmission than of the symptoms of HPV infection. Most mothers and daughters correctly indicated that “HPV can be transmitted through sexual intercourse” (68.2% vs. 75.9%), and that “having sex with multiple sexual partners increases the risk of HPV infection” (75.3% vs. 77.6%). However, more mothers than daughters knew that “having sex before the age of 16 increases the risk of HPV infection” (61.8% vs. 49.4%), with the difference being statistically significant (*p* = 0.017).

Generally, fewer mothers than daughters correctly answered those items related to the HPV vaccine and cures for HPV. However, more daughters than mothers answered correctly that the following statements were false: “currently, there is a cure to eliminate the HPV virus” (39.4% vs. 52.9%, *p* = 0.007), and “HPV can be cured with antibiotics” (54.1% vs. 70.0%, *p* = 0.001), and the difference was statistically significant.

### Attitudes towards HPV infection, cervical cancer, and the HPV vaccine

Table 3 shows the attitudes of mothers and adolescent daughters towards HPV infection and the HPV vaccine, and comparisons between them that were made using Chi square tests.

**Table 3:** Attitudes towards HPV infection and the HPV vaccine (N = 170 mother–daughter dyads)

	Mothers (n = 170)		Daughters (n = 170)		χ^2^	*p* value
	n	(%)	n	(%)		
Perception of the risk and severity of HPV infection and cervical cancer for daughter						
Presence of health threat if infected with HPV	163	95.9	169	99.4		0.067^a^
Presence of health threat if getting cervical cancer	163	95.9	169	99.4		0.067^a^
Chance of being infected with HPV in lifetime without the HPV vaccine	148	87.1	162	95.3	6.457	0.011*
Chance of getting cervical cancer in the future without the HPV vaccine	151	88.8	162	95.3	5.227	0.022*
Stigmatization						
People will be stigmatized as promiscuous for having been infected with HPV	24	14.1	23	13.5	0.032	0.858
Daughters (or the girls themselves) will be stigmatized as promiscuous if they have received HPV vaccination	20	11.8	6	3.5	8.254	0.004**
Having received the HPV vaccine will encourage an early sexual debut	26	15.3	21	12.4	0.652	0.419
Perception that a person is too young to receive a vaccine associated with sexual transmitted illness						
Daughters (or the girls themselves) are too young to receive a vaccine for an STI like HPV	65	38.2	43	25.3	6.769	0.009**
Safety and effectiveness of the HPV vaccine						
The HPV vaccine is safe	124	72.9	137	80.6	1.972	0.160
Can prevent most genital warts	114	67.1	126	74.1	1.041	0.307
Can prevent most HPV infections	123	72.4	129	75.9	0.222	0.637
Can prevent cervical cancer	131	77.1	134	78.8	0.007	0.932
Accessibility of the HPV vaccine						
Costs more than the parents can afford	115	67.6	108	63.5	1.070	0.301
Difficult to find a provider or clinic that has the vaccine available	66	38.8	60	35.3	0.579	0.447
Agrees that the government should subsidize the full cost of HPV vaccination	139	81.8	147	86.5	0.962	0.327

Individual items may or may not add up to the total due to some missing data

^a^For cells with less than 5 counts, Fisher’s exact test was adopted

* *p* ≤ 0.05; ** *p* ≤ 0.01

Over 95% of both the mothers and daughters perceived a health threat if they were to be infected with HPV (95.9% vs. 99.4%) or develop cervical cancer (95.9% vs. 99.4%). However, more daughters than mothers perceived that without the HPV vaccine, they would stand a greater chance of being infected with HPV in their lifetime (95.3% vs. 87.1%, *p* = 0.011) and getting cervical cancer in the future (95.3% vs. 88.8%, *p* = 0.022).

Few mothers and daughters considered that “people would be stigmatized as promiscuous if they become infected with HPV” (14.1% vs. 13.5%), or that “having received the HPV vaccine will encourage an early sexual debut” (15.3% vs. 12.4%), with no statistically significant difference observed between the two groups. However, more mothers than daughters were concerned that their daughters would be stigmatized as promiscuous if they received HPV vaccination (11.8% vs. 3.5%, *p* = 0.004). Mothers were also more likely to perceive that their daughters were too young to receive a vaccine for a sexually transmitted infection like HPV (38.2% vs. 25.3%, *p* = 0.009).

With regard to the safety and effectiveness of the HPV vaccine, the majority of the mothers and daughters considered that the “HPV vaccine is safe” (72.9% vs. 80.6%) and “can prevent most genital warts” (67.1% vs. 74.1%), “most HPV infections” (72.4% vs. 75.9%), and “cervical cancer” (77.1% vs. 78.8%). Although in general, there were no statistically significant differences between the two groups, slightly more daughters than mothers agreed that the HPV vaccine was safe and effective.

On the issue of the accessibility of the HPV vaccine, over 60% of the mothers and daughters agreed that the HPV vaccine costs more than they (or their parents) can afford (67.6% vs. 63.5%), but fewer reported that it was difficult to find a provider or clinic that has the vaccine (38.8% vs. 35.3%). The majority of mothers and daughters agreed that the government should subsidize the full cost of the vaccine (81.8% vs. 86.5%). No statistically significant differences were observed between the two groups.

### Reasons for the acceptance or non-acceptance of HPV vaccination

Table 4 shows the comparison that was made, using a Chi square test, between the mothers and daughters on their reasons for accepting or not accepting HPV vaccination. Only about one-third of the mothers (35.3%) and daughters (36.5%) indicated that they would be “very likely” to accept HPV vaccination for their daughters or for themselves if the vaccines were available for free. No significant differences between the two groups were observed.

**Table 4:** Reasons for accepting and not accepting HPV vaccination (N = 170 dyads)

	Mothers (n = 170)		Daughters (n = 170)		χ^2^	*p* value
	n	(%)	n	(%)		
Acceptance of HPV vaccination (participants who were “very likely” to accept vaccination)	60	35.3	62	36.5	0.051	0.821
Reasons for accepting HPV vaccination						
The HPV vaccine is effective at preventing cervical cancer	140	82.4	154	90.6	2.440	0.118
Authoritative reassurances about safety and efficacy are given	134	78.8	129	75.9	0.621	0.431
The HPV vaccine is recommended by doctors	129	75.9	128	75.3	0.193	0.660
The HPV vaccine is recommended by the school or government	112	65.9	96	56.5	3.919	0.048*
Daughters asked to have the HPV vaccination	98	57.6	88	51.8	1.709	0.191
Other parents are getting their daughters vaccinated against HPV	72	42.4	66	38.8	0.582	0.445
Reasons for not accepting HPV vaccination						
Do not want daughters (or themselves) to be stigmatized as promiscuous	64	37.6	7	4.1	64.486	<0.001**
The side-effects of the HPV vaccine are unknown since the vaccine is relatively new	110	64.7	110	64.7	0.007	0.933
The HPV vaccine costs more than they (their parents) can pay	106	62.4	109	64.1	0.039	0.843
Have inadequate information about the vaccine to make a decision	91	53.5	102	60.0	1.262	0.261
The safety of the vaccine has not yet been confirmed	97	57.1	82	48.2	3.082	0.079
Consider their daughters (or themselves) still too young for the vaccine	79	46.5	79	46.5	0.003	0.955
The HPV vaccine causes short-term problems like fever or discomfort	82	48.2	73	42.9	1.684	0.194
The HPV vaccine might cause lasting health problems	70	41.2	59	34.7	1.975	0.160
The HPV vaccine often cause dizziness or nausea	66	38.8	63	37.1	0.286	0.593
The HPV vaccine often causes severe pain at the injection site	58	34.1	56	32.9	0.167	0.683
No one had ever informed them about the HPV vaccine	55	32.4	64	37.6	1.001	0.317
Daughters (or parents) did not agree to receive the vaccine	52	30.6	40	23.5	2.320	0.128

Individual items may or may not add up to the total due to some missing data

* *p* ≤ 0.05; ** *p* ≤ 0.01

A large percentage of mothers and daughters considered the following to be their main reasons for accepting HPV vaccination: “The HPV vaccine is effective at preventing cervical cancer” (82.4% vs. 90.6%), “Authoritative assurances are given about safety and efficacy” (78.8% vs. 75.9%), and “The HPV vaccine is recommended by doctors” (75.9% vs. 75.3%). Significantly more mothers than daughters indicated acceptance if the “HPV vaccine is recommended by the school or the government” (65.9% vs. 56.5%, *p* = 0.048). Only a little more than half of the mothers and daughters “asked to be vaccinated against HPV” (57.6% vs. 51.8%). Less than half indicated that the belief that “other parents are getting their daughters vaccinated against HPV” (42.4% vs. 38.8%) was a reason for accepting vaccination.

Many reasons were provided for not accepting HPV vaccination. The major reasons both mothers and daughters gave for not accepting HPV vaccination included: “The side-effects of the HPV vaccine are unknown since the vaccine is relatively new” (64.7% vs. 64.7%), and “The HPV vaccine costs more than I can afford” (62.4% vs. 64.1%). There were statistically significant differences between the mothers and daughters in agreeing that the following was a reason for their non-acceptance: “Do not want my daughter/or myself to be stigmatized as promiscuous” (37.6% vs. 4.1%, *p* < 0.001).

### Factors predicting acceptance of HPV vaccination

Table 5 shows an analysis using binary logistic regression of the factors predicting acceptance of the HPV vaccine. Two separate analyses were conducted with the dependent variables of the acceptance of mothers and of daughters. The independent variables included for analysis were all of the items listed in Tables 2, 3 and 4.

**Table 5:** Factors predictive of acceptance of the HPV vaccine, analyzed by binary logistic regression

	B	SE	OR	95% CI	Sig.
Dependent variable: acceptance of mothers (n = 60)					
The HPV vaccine is safe	2.315	0.720	10.126	2.468–41.542	0.001
Daughters asked to have the HPV vaccination	2.150	0.576	8.582	2.777–26.518	<0.001
Difficult to find a provider or clinic that has the vaccine available	2.060	0.585	7.848	2.493–24.705	<0.001
The HPV vaccine might cause lasting health problems	1.762	0.577	5.825	1.879–18.058	0.002
Do not want daughters to be stigmatized as promiscuous	–1.987	0.575	0.137	0.044–0.423	0.001
Consider their daughters still too young for the vaccine	–1.104	0.465	0.332	0.133–0.824	0.018
Dependent variable: acceptance of daughters (n = 62)					
Can prevent most HPV infections	1.836	0.602	6.274	1.927–20.423	0.002
The side-effects of the HPV vaccine are unknown since the vaccine is relatively new	1.402	0.509	4.062	1.499–11.006	0.006
The HPV vaccine costs more their parents can pay	1.356	0.503	3.880	1.448–10.397	0.007
The HPV vaccine is effective at preventing cervical cancer	–2.059	0.701	0.128	0.032–0.504	0.003
The safety of the vaccine has not yet been confirmed	–1.548	0.489	0.213	0.082–0.554	0.002
The HPV vaccine might cause lasting health problems	–1.051	0.522	0.349	0.126–0.971	0.044

*SE* standard error, *Sig.* significance, *CI* confidence interval

The results indicated that when considering whether or not to accept HPV vaccination for their adolescent girls, the predictive factors of the mothers’ acceptance of HPV vaccination were: “the HPV vaccine is safe” (OR = 10.13, 95% CI 2.47–41.54, *p* = 0.001), and “Daughters asked to receive HPV vaccination” (OR = 8.58, 95% CI 2.78–26.52, *p* < 0.001). However, the mothers who accepted vaccination for their daughters also expressed concern that it is “difficult to find a provider or clinic that has the vaccine available” (OR = 7.85, 95% CI 2.49–24.71, *p* < 0.001), and “The HPV vaccine might cause lasting health problems” (OR = 5.83, 95% CI 1.88–18.06, *p* = 0.002). Mothers were less likely to accept vaccination when they “do not want their daughters to be stigmatized as promiscuous” (OR = 0.14, 95% CI 0.04–0.42, *p* = 0.001), and “consider their daughters still too young for the vaccine” (OR = 0.33, 95% CI 0.133–0.82, *p* = 0.028).

The factors considered by the daughters were different from those of the mothers. The factor predicting a high likelihood that the daughters would accept HPV vaccination was their belief that the HPV vaccine “can prevent most HPV infections” (OR = 6.274, 95% CI 1.927–20.423, *p* = 0.002). However, the daughters were also concerned that the “side-effects of the HPV vaccine are unknown since the vaccine is relatively new” (OR = 4.062, 95% CI 1.499–11.006, *p* = 0.006), and “the HPV vaccine costs more than my parents can pay” (OR = 3.880, 95% CI 1.448–10.397, *p* = 0.007). Those daughters who accepted the vaccine did not take into consideration the fact that “The HPV vaccine is effective at preventing cervical cancer” (OR = 0.128, 95% CI 0.032–0.504, *p* = 0.003) as a reason for their acceptance. Daughters who accepted the vaccine were also less likely to be concerned that “the safety of the vaccine has not yet been confirmed” (OR = 0.213, 95% CI 0.082–0.554, *p* = 0.002), and “the HPV vaccine might cause lasting health problems” (OR = 0.349, 95% CI 0.126–0.971, *p* = 0.044).

## Discussion

### Demographic characteristics of the mothers and their daughters

The percentage of mothers (76.4%) in this study who had attained an educational level of secondary school or above is similar to the figure for women in Hong Kong in general [30]. However, they had a slightly lower family income than the general population in Hong Kong [31]. The results also show that 55.3% of the mothers had ever had a Pap smear test, which is slightly lower than the finding of 62.9% of women aged 18–64 in Hong Kong who had ever had a Pap smear test reported by the Centre for Health Protection [32].

The majority of the adolescent daughters (82.4%) had received all of the childhood vaccines recommended in the Hong Kong Childhood Immunization Program. However, only 1.8% had received the HPV vaccination. In Hong Kong, the HPV vaccine is not included in the immunization program for young children. The uptake rate for the vaccine is lower than the 2.4% reported in another study in Hong Kong among secondary school girls [33], and much lower than that reported in Western countries, of between 17 and 81% [34]. However, the portion of adolescent daughters who reported having had sexual experience (15.9%) was higher than the 7.4% reported among general secondary school girls in Hong Kong in 2011 [35].

### Knowledge of HPV and the HPV vaccine

Both the mothers and adolescent daughters in this study showed a similar or even higher level of knowledge of HPV infection and the HPV vaccine than those in previous studies. A previous study reported that adolescents had limited knowledge of the symptoms of HPV infection [36]. The percentage of mothers and adolescent daughters in this study who knew that itching or bleeding warts are a symptom of HPV infection is comparable to the finding in a systematic review that about 55% of young adults and adolescents knew that genital warts are a symptom of HPV infection [13].

The level of knowledge of the mothers (67.6%) and adolescents (74.1%) about the link between HPV infection and cervical cancer is higher than that reported in previous studies in Hong Kong, where it was found that only 33% of mothers were knowledgeable about the link between HPV and cervical cancer [9]. This was also the case in the Netherlands and Brazil, where few parents (14%) and young women (9.8%) were aware of the causal relationship between HPV and cervical cancer [37, 38].

This finding that the mothers (68.2%) and daughters (75.9%) in this study demonstrated relatively good knowledge of the transmission of HPV is consistent with that in other studies [12, 19]. Another study in Hong Kong also reported similar percentages, with about 60–77% of women found to be knowledgeable about this risk of having multiple sexual partners [9]. More mothers (61.8%) in this study knew that sexual intercourse before the age of 16 is a risk factor for HPV infection than the percentages reported in another study in Hong Kong, where only 43.6% of the women knew that early sexual intercourse is a risk factor for HPV infection [9].

When asked about the HPV vaccine and cures, the adolescent daughters demonstrated a higher level of knowledge than their mothers. This is an improvement from a previous study conducted in Hong Kong that reported that as many as 87% of the women mistakenly thought that HPV could be eliminated by treatment [19]. However, the percentages are still much lower than that of a study conducted in the United States, which found that over 80% of the young women could correctly state that HPV is not curable by antibiotics [12].

Both the mothers and daughters in this study demonstrated a better understanding and awareness of the HPV vaccine than those in previous studies. Their awareness of the HPV vaccine is higher than that found in other studies conducted earlier in Hong Kong, in which it was reported that no more than half of the women had ever heard of vaccination against HPV and cervical cancer [19, 23]. The finding is consistent with another study that found that 76% of Hong Kong women knew that it is necessary to undergo cervical screening even after receiving the HPV vaccination [19].

To conclude, the increased knowledge demonstrated in this study of the symptoms and causal relationship of HPV, HPV vaccines, and cervical cancer by the mothers and daughters in Hong Kong may be due to their increased exposure to related promotional messages from the media regarding the illness and vaccines in Hong Kong in recent years.

### Attitude towards HPV and the HPV vaccine

The results show that the majority of mothers and daughters would perceive it as a health threat if their daughters (or they themselves) were to be infected with HPV or diagnosed with cervical cancer. This is a very different picture from that painted in a previous systematic review, where it was reported that fewer than half of adolescents and young women (21–46%) perceived themselves to be at risk of HPV infection [13]. It was suggested that the adolescents’ lack of experience with sexual activity may have contributed to their perception that they are at a low risk of HPV infection, as only 8% of Chinese adolescent girls were reported to be sexually active [14]. The increase in the perceived risk of HPV infection among the daughters in the present study may due to the reported increase in the percentage of daughters who have had sexual experience (15.9%).

The percentage of Chinese mothers (11.8%) who were concerned that their daughters might be considered promiscuous was higher than in Australia, where the figure was 5% of parents [39]. The proportion of mothers (15.3%) who believed that having received the HPV vaccine will encourage an early sexual debut was slightly higher than that reported in other studies among parents in Hong Kong, Vietnam, the US, and England (2.1–12%) [9, 13, 21, 39]. This may due to the Hong Kong mothers’ belief that the vaccine would encourage promiscuity and that their children are too young to be sexually active [21].

The majority of both mothers and daughters perceived the HPV vaccine to be safe and effective at preventing most genital warts, HPV infection, and cervical cancer. This result is similar to the findings of a previous study indicating that a high percentage of people believed that the HPV vaccine would be effective [40]. However, the cost of the HPV vaccine is a major concern, as over 60% of the mothers and daughters reported that the HPV vaccine costs more than they (or their parents) can afford. Likewise, they agreed that the government should subsidize the full cost of HPV vaccination.

### Reasons for the acceptance or non-acceptance of HPV vaccination

The rates of acceptance of HPV vaccination (35.3% of mothers and 36.5% of daughters) were slightly higher than the 32% reported in a previous study conducted by Chan et al. [9] in Hong Kong. These rates, however, are still much lower than those reported in Western countries such as California, the US (75%), and Finland (86%) [10, 11].

In our study, the safety and effectiveness of the HPV vaccine in preventing infection and cervical cancer were the main reasons why both the mothers and their daughters were open to accepting HPV vaccination. This is supported by the findings in other studies indicating that people are more likely to accept HPV vaccination when they believe that the vaccine is effective against cervical cancer [13, 29]. Other studies have also reported that having trustworthy evidence of the effectiveness of the HPV vaccine was associated with parental acceptance of vaccination for their children [21], and that a recommendation from doctors was important in making the decision to be vaccinated against HPV [13]. In this study, the mothers and their adolescent daughters also indicated that the school’s or government’s recommendation that adolescent girls be vaccinated against HPV was instrumental in their acceptance of HPV vaccination.

Both the mothers and daughters in this study shared the concern that since the vaccine is relatively new, its possible side-effects are unknown. The side-effects of the HPV vaccine were one reason why they did not accept HPV vaccination. This result is consistent with other studies indicating that parents were concerned about the possible side-effects of the vaccine [39], and that those who expressed suspicion of the safety of the vaccine were less likely to accept HPV vaccination [21].

The participants in this study believed that the HPV vaccine costs more than they can afford, and indicated that this was a reason for their not accepting the vaccination. This concurs with a previous study in which it was reported that the cost of the vaccine decreased the intention of the participants to be vaccinated against HPV [14]. However, the same author argued in a later study that mothers considered the cost of the vaccination to be “reasonable” for the protection that it provided against a potentially life-threatening disease [19]. It is speculated that the socio-economic status of families may contribute to their different perceptions of the cost of the vaccine, and thus be a factor in their (non)acceptance of HPV vaccination.

Not wanting their daughters (or themselves) to be stigmatized as promiscuous was a reason for their non-acceptance of vaccination, but the difference between the mothers and their daughters on this issue was significant. The result is consistent with another study in which it was also reported that parental worries about promiscuity arising from vaccination acted as a barrier to vaccination [24].

### Factors predicting acceptance of the HPV vaccine

Among all of the considerations, the factors predictive of whether mothers would accept HPV vaccination for their daughters were that the vaccine is considered safe and their daughters made the request to be vaccinated. It is obvious that the mothers cared about the health of their daughters and respected their daughters’ decision. In other words, the acceptance of HPV vaccination among the daughters also contributed to the acceptance by the mothers of the HPV vaccine for their daughters.

Mothers who accepted the HPV vaccine were also more likely to state that it was difficult to find a clinic that provides vaccination against HPV, and to express the view that the HPV vaccine might cause lasting health problems. The low accessibility and adverse effects of the vaccine were concerns that the mothers had relating to the HPV vaccine. On the other hand, mothers who worried that their daughters would be stigmatized as promiscuous and who considered their daughters too young to be vaccinated were less likely to accept the HPV vaccination for their adolescent daughters.

Daughters who accepted the HPV vaccine were more likely to believe that “The HPV vaccine can prevent most HPV infections.” Interestingly, consideration of the effectiveness of the vaccine against cervical cancer was not a factor in their decision to be vaccinated. The adolescent girls were more concerned about preventing HPV infection than about the “distant” health problem of developing cervical cancer.

The adolescent daughters who accepted the vaccination were concerned about the side-effects and potential health problems caused by the HPV vaccine. This finding is consistent with another study in Hong Kong that found that the majority of the adolescent girls that were interviewed decided to be vaccinated on the condition that assurances about the safety and efficacy of the vaccine be given, and before the issue of the high cost of the HPV vaccine was mentioned [14].

## Limitations

This study has its limitations in terms of the study design, sampling method, and sample size. The cross-sectional and non-experimental design of this study contributed to the limitation that no direct causal relationship between the variables could be made. With the limitation in the sampling method, which involved recruiting participants from only one secondary school, the results of the study may not be generalizable to the population as a whole. The participants in this study were from somewhat lower-income families. A sample of only 170 mother and daughter pairs was recruited, and only a small number of participants had characteristics that might influence their acceptance of HPV vaccination, such as a history of cervical cancer in the case of the mothers and sexual experience in the case of the daughters.

Various factors related to vaccine acceptance were examined in the study; there could have been other potential variables associated with the mothers’ and daughters’ acceptance of vaccination for adolescent girls that were not explored in this study.

## Conclusions

The findings of this study provide healthcare professionals with a better understanding of the differences between mothers and adolescent girls in their knowledge, attitude, and potential factors associated with acceptance of the HPV vaccine. The results of this study showed that both the mothers and their adolescent daughters had relatively little knowledge of HPV infection, but better awareness of the causal relationship between HPV and cervical cancer. Mothers were more aware of the risk of early sexual intercourse as a factor in HPV infection, while adolescent daughters were more knowledgeable about the HPV vaccine and cures for HPV.

Daughters were more likely to consider themselves to be at risk of contracting an HPV infection and cervical cancer without the HPV vaccine, while mothers were more concerned that their daughters would be stigmatized as promiscuous if they were vaccinated against HPV, and felt that their daughters were too young to receive the vaccine. The safety and effectiveness of the HPV vaccine were the main reasons and also the predictive factors behind the decision of both mothers and daughters to accept HPV vaccination for adolescent girls. However, the low accessibility and possible lasting health problems caused by the vaccine may be barriers to the acceptance of the vaccine by mothers. Potential barriers also include the unknown side-effects and high cost of the HPV vaccine.

## Implications and recommendations

Generally, both mothers and daughters had a relatively good level of knowledge of HPV infection, transmission, and the HPV vaccine, but held the attitude that HPV is associated with promiscuity and that adolescents are too young to receive the vaccine. It has been suggested that in promoting HPV vaccination and the prevention of cervical cancer, one strategy should include providing health education not only to increase knowledge but also to minimize the stigma and negative attitudes towards sexually transmitted HPV infection [23]. Education on HPV infection, the risk of early engagement in sexual intercourse, and HPV treatment should also be provided in schools to adolescents. Health professionals should focus on eliminating concerns and worries about the stigmatizing effect arising from vaccination.

The safety and effectiveness of the vaccination were found in this study to be the most influential facilitators of acceptance of the HPV vaccine for adolescent girls. Adolescents should be given assurance of the safety of the HPV vaccine and the importance of the early prevention of HPV infection.

The barriers to vaccination that were identified were accessibility and the cost of HPV vaccines. In societies where coverage of HPV vaccination is low, the provision of HPV vaccines in school-based vaccination programs may be a way of addressing the problem of accessibility, as the mothers in this study stated that they would be more likely to accept vaccination for their daughters if it is recommended by the school or the government. It was also been reported that most parents preferred universal school-based vaccination to other approaches to vaccination [21]. Another study also revealed that support from the government, doctors, or schools is considered key to promoting vaccination against HPV infection [23]. The government should also consider providing the vaccine free of charge or giving partial subsidies to adolescents. It is expected that school-based programs will cover at least 90% of 12-year-old adolescent girls [41]. The support of the government and the provision of school-based vaccination programs will not only increase the accessibility of the associated vaccination, but also reduce the stigma of receiving it.

An evaluation study should be conducted to assess the coverage of school-based vaccination programs and the long-term effects of preventing HPV infections and cervical cancer. As the Centers for Disease Control and Prevention [42] have recommended that boys should also receive the vaccination, a further study should be conducted of the views of parents and boys on HPV vaccination and its long-term effects.

## Abbreviations

ACIP: Advisory Committee on Immunization Practices
HKMA: Hong Kong Medical Association
HPV: human papillomavirus
SIL: squamous intraepithelial lesions
STI: sexually transmitted infections
WHO: World Health Organization
